# Enhancing the Differentiation between Intestinal Behçet’s Disease and Crohn’s Disease through Quantitative Computed Tomography Analysis

**DOI:** 10.3390/bioengineering10101211

**Published:** 2023-10-17

**Authors:** Yuanqiu Li, Ziman Xiong, Yuchen Jiang, Yaqi Shen, Xuemei Hu, Daoyu Hu, Zhen Li

**Affiliations:** Department of Radiology, Tongji Hospital, Tongji Medical College, Huazhong University of Science and Technology, Wuhan 430030, China; m202176423@hust.edu.cn (Y.L.); zmxiong@hust.edu.cn (Z.X.); jiangyuchen@hust.edu.cn (Y.J.); xmhu@hust.edu.cn (X.H.); daoyuhu@hust.edu.cn (D.H.); zhenli@hust.edu.cn (Z.L.)

**Keywords:** intestinal Behçet’s disease, Crohn’s disease, computed tomography enterography, body composition

## Abstract

Behçet’s disease (BD) behaves similarly to Crohn’s disease (CD) when the bowel is involved. Computed tomography enterography (CTE) can accurately show intestinal involvement and obtain body composition data. The objective of this study was to evaluate whether CTE could improve the ability to distinguish between intestinal BD and CD. This study evaluated clinical, laboratory, endoscopic, and CTE features on first admission. Body composition analysis was based on the CTE arterial phase. The middle layers of the L1–L5 vertebral body were selected. The indicators assessed included: the area ratio of visceral adipose tissue (VAT)/subcutaneous adipose tissue (SAT) (VSR) in each layer, the total volume ratio of VAT/SAT, the quartile of VAT attenuation in each layer and the coefficient of variation (CV) of the VAT area for each patient was also calculated. Two models were developed based on the above indicators: one was a traditional model (age, gender, ulcer distribution) and the other was a comprehensive model (age, gender, ulcer distribution, proximal ileum involvement, asymmetrical thickening of bowel wall, intestinal stenosis, VSR_L4_, and CV). The areas under the receiver operating characteristic (ROC) curve of the traditional (sensitivity: 80.0%, specificity: 81.0%) and comprehensive (sensitivity: 95.0%, specificity: 87.2%) models were 0.862 and 0.941, respectively (*p* = 0.005).

## 1. Introduction

Behçet’s disease (BD) is a chronic multisystem vascular inflammatory disease. The disease was first described as a separate entity by Huluci Behçet in 1937. The regions along the ancient silk road, from the Mediterranean Basin to East Asia, have the highest prevalence of BD and generally experience more severe conditions [1]. In contrast, the prevalence is lower in North American and Northern European countries [2].

BD was characterized by recurrent aphthous oral ulcers as well as a variety of systemic manifestations, such as genital ulcers, characteristic skin lesions, and lesions of the eye, nervous system, blood vessels, joints, and gastrointestinal tract. The incidence of gastrointestinal involvement in BD patients was estimated to range from 1.4% to 60% [3]. Gastrointestinal manifestations in BD usually appeared 4.5–6 years later than oral ulcers. In certain instances, intestinal lesions can predate extraintestinal manifestations [4]. The most widely used and recognized diagnostic criteria of BD is the International Study Group (ISG) Criteria published in 1990 based on parenteral manifestations and pathergy test [5]. Misdiagnosis often occurs when BD primarily manifests with gastrointestinal symptoms.

BD and Crohn’s disease (CD) can result in overlapping intestinal and extraintestinal manifestations, often leading to misdiagnosis. Expert endoscopists have characterized the distinctive ulcers found in intestinal BD as large, ovoid, and deep, with well-defined margins. These ulcers are primarily located in the ileocecal region [6]. Therefore, based on colonoscopy findings and extraintestinal systemic manifestations, a study in South Korea proposed a diagnostic criteria for intestinal BD [6]. Typical endoscopic findings in CD include discontinuously distributed longitudinal ulcers, cobblestone-like appearance, and/or small longitudinally arranged aphthous ulcers [7]. Nevertheless, accurate identification continues to heavily depend on the proficiency of the executing physician. The strong alignment between endoscopy and computed tomography (CT) imaging, however, opens a promising pathway for these unique characteristics to be discernible in CT scans as well. Computed tomography enterography (CTE) is a newly developed technique that can accurately display mucosal involvement, intestinal wall thickening, and parenteral complications. The ileocecal region is commonly affected, showing polypoid thickening with obvious enhancement in intestinal BD patients [3,8]. Intestinal fistula, stenosis, and abscess were less prevalent in patients with intestinal BD than in those with CD [9]. Additionally, in CD patients, intestinal wall thickening and enhancement are typically more severe on the mesenteric side of the bowel wall [10].

Chronic inflammatory diseases are accompanied by changes in body composition. CD is characterized by malnutrition, resulting in the redistribution of fat mass [11]. Mesenteric fat accumulation is one of the specific features of CD. Imaging biomarkers based on CT such as the visceral fat area can be used to reflect surgical tissue fibrosis [12] and predict the postoperative course [13], disease activity, and prognosis [14] of CD patients. On the other hand, the study of body composition in intestinal BD is still limited. Existing research has qualitatively indicated that intestinal BD patients may exhibit mesenteric fibrofatty proliferation [3,15]. However, there is currently a lack of quantitative studies investigating abdominal fat in intestinal BD patients.

The aim of this study was to evaluate whether image characteristics and body composition analysis based on CTE can improve the ability to distinguish between intestinal BD and CD compared with a combination of clinical symptoms and endoscopic features.

## 2. Materials and Methods

### 2.1. Patients

This study was approved by local research ethics committees, and informed consent was waived. A systematic review of the electronic medical record system in our institution from January 2015 to October 2022 was performed to search for patients meeting the requirements.

The diagnosis of intestinal BD patients followed the criteria proposed by Cheon J.H. et al. [6] or multidisciplinary team (MDT) discussion and ruled out the diagnosis of intestinal tuberculosis. Definite, probable, and suspected intestinal BD groups according to Cheon’s study were enrolled. The diagnosis of CD patients followed the World Gastroenterology Organization Global Guidelines [16]. Exclusion criteria were as follows: (1) without available endoscopy and CTE; (2) underwent previous intestinal surgery; (3) patients received immunotherapy and hormone therapy for other reasons within 6 months; (4) concomitant malignancy; and (5) aged <18 at the first admission.

### 2.2. Assessment Items

Clinical data, laboratory tests, endoscopic data, and CTE features on first admission were evaluated.

Clinical data were as follows: age, sex, disease course (from onset of gastrointestinal symptoms to the first admission), abdominal pain, diarrhea, fever, weight loss, gastrointestinal bleeding (bloody stool or positive fecal occult blood), oral ulcers, genital ulcers, perianal lesions, and intestinal symptoms as primary manifestation.

Laboratory indicators included: C-reactive protein (CRP), erythrocyte sedimentation rate (ESR), albumin, and hemoglobin.

The intestinal segment was divided into jejunum, proximal ileum, terminal ileum, cecum, ascending colon, transverse colon, descending colon, sigmoid colon, and rectum. Endoscopic findings included: focal ulcer distribution (limited to one segment or two contiguous segments) or segmental distribution (discontinuous ulcer with more than two segments) [17], longitudinal ulcer, and ileocecal valve deformity. Additionally, the presence of noncaseating granuloma during endoscopic biopsy was also documented.

**Figure 1:**
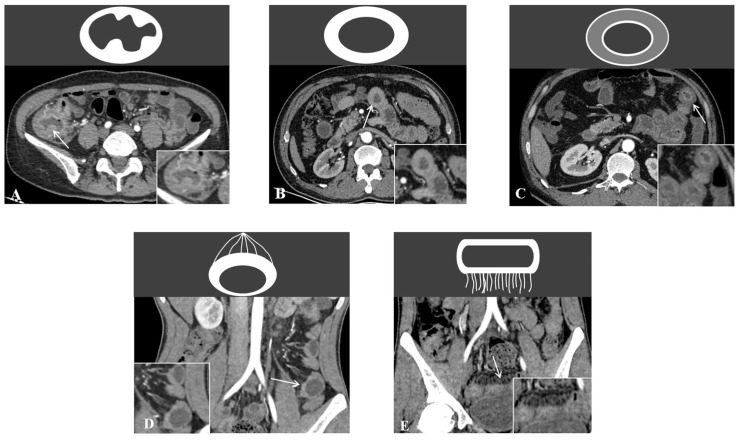
Illustrative diagram of imaging assessment features. The white arrows indicate: (**A**) polypoid bowel wall thickening; (**B**) homogeneous bowel wall thickening and homogeneous enhancement pattern; (**C**) layered enhancement pattern; (**D**) asymmetrical thickening of bowel wall; and (**E**) comb sign.

The image features were blindly evaluated by two senior radiologists. According to previous literature [3,18] and the clinical experience of our research group, imaging features selected included (Figure 1): a polypoid or homogeneous pattern of bowel wall thickening (if any intestinal segment showed polypoid thickening, it was recorded as polypoid thickening; otherwise, it was recorded as homogeneous thickening); a layered or homogeneous enhancement pattern (if any intestinal segment showed layered enhancement, it was recorded as layered enhancement; otherwise, it was recorded as homogeneous enhancement); intestinal segment involvement, asymmetrical thickening of bowel wall, comb sign, intestinal stenosis (localized lumen stenosis and bowel wall thickening with or without prestenosis dilation [19]), and penetration (sinus, fistula, abscess).

### 2.3. Quantification of Body Composition Parameters Based on CTE

**Figure 2:**
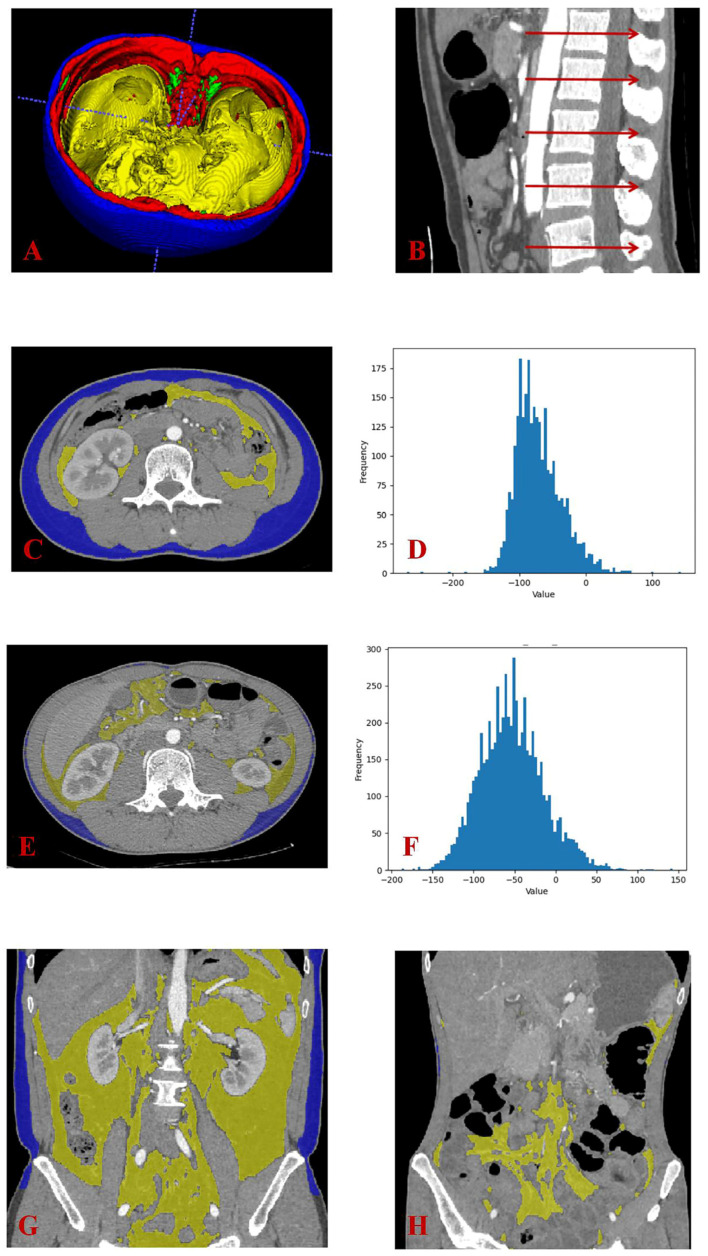
An automatic segmentation algorithm was used to obtain quantitative body composition data based on the arterial phase of CTE. (**A**) This was a three-dimensional display of the automatic segmentation result. Blue represents SAT; red represents skeletal muscle; green represents intermuscular fat; yellow represents VAT. (**B**) We evaluated quantitative data at the intermediate layer of the L1–L5 vertebral body. (**C**,**D**) These two pictures show the VAT and SAT area and the attenuation of VAT at L3 of intestinal BD patient. (**E**,**F**) These two pictures showed the VAT and SAT area and attenuation of VAT at L3 of CD patient. (**G**,**H**) These two pictures show the coronal views of CTE for the intestinal BD patient and CD patient, respectively. It can be observed that VAT was primarily concentrated in the lower abdomen in the CD patient, while the distribution of VAT appeared more uniform in the intestinal BD patient. CTE, computed tomography enterography; SAT, subcutaneous adipose tissue; VAT, visceral adipose tissue; BD, Behçet’s disease; CD, Crohn’s disease.

The quantitative data for body composition were obtained from an automatic segmentation algorithm based on the arterial phase of CTE (Figure 2). The code can be obtained at https://github.com/CharelBIT/nnUNet-modify (accessed on 21 May 2023). The area of subcutaneous adipose tissue (SAT) and visceral adipose tissue (VAT), as well as the attenuation quartile (25% quartile, median, 75% quartile) of VAT, were measured at the intermediate layer of the first lumbar (L1) to the L5 vertebral body (Figure 2). The volume of SAT and VAT between L1 and L5 was also recorded. Referring to previous research [20], the area ratio of the VAT/SAT (VSR) of each level and the volume ratio of VAT/SAT (VSR_volume_) were calculated to reflect the proportion of VAT. A coefficient of variation (CV) was obtained by calculating the mean value and standard deviation of the VAT area of 5 layers for each patient to reflect the distribution of VAT.

### 2.4. Imaging Technique

CTE was conducted following the recommended technique outlined in the first joint consensus statement on inflammatory bowel disease by ESGAR/ESPR, published in 2017. [21]. The scan was performed after 4–6 h fasting. The contrast agent was iopromide (Ultravist 370, Bayer Schering Pharma, Berlin, Germany) and iodixanol (Visipaque 320, General Electric Pharmaceutical Industry Co., Ltd., Shanghai, China). Contrast agent was injected intravenously at a rate of 3.5 mL/s, followed by a 20 mL saline flush. The total amount of contrast agent was calculated according to the body weight at 1~1.1 mL/kg.

### 2.5. Statistical Analysis

Statistical analyses were performed using SPSS version 25.0, MedCalc Statistical Software version 20.022 and Python software version 3.11.4 (Python software Foundation). Data were presented as the mean ±  standard deviation (SD) for normal data and median (25% quartile, 75% quartile) for non-normal data. Categorical variables were analyzed by χ^2^ test. Continuous variables were analyzed by *t*-test or Mann–Whitney U test. All indicators with *p* < 0.05 were statistically significant. Kappa statistics were used to assess the agreement between two senior radiologists. The degree of agreement was determined as follows: ≤0.20, slight agreement; 0.21–0.40, fair agreement; 0.41–0.60, moderate agreement; 0.61–0.80, good agreement; and >0.80, excellent agreement. Binary logistics regression was used for univariable analysis for the variables of interest (when selecting variables of interest for univariable analysis, the colonic segments and rectum within the image features were combined into a single variable called the colorectum). Variables with *p* < 0.1 were enrolled for multivariable analysis to build two identification models (traditional model: clinical data combined with endoscopic features; comprehensive model: clinical data, endoscopic features and CTE features including quantitative index of body composition). Receiver operating characteristic (ROC) curves were drawn to compare the efficacy of the two models. Then, we validated these two models using leave-one-out cross-validation on the same set of patients.

## 3. Results

**Figure 3:**
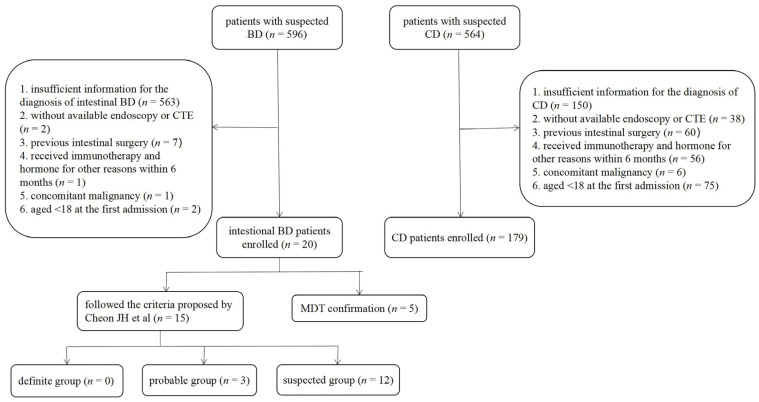
The flow chart of the study. BD, Behçet’s disease; CD, Crohn’s disease; CTE: computed tomography enterography; MDT, multidisciplinary team.

Twenty intestinal BD patients and 179 CD patients were enrolled in this study. The flow chart is presented in Figure 3.

### 3.1. Clinical Date

The clinical information, laboratory results, and endoscopic characteristics of enrolled intestinal BD and CD patients are shown in Table 1. The majority of the two groups was male, and the proportion of male patients in CD patients was higher than that in intestinal BD patients (*p* = 0.025). Intestinal BD patients were older than CD patients (38.00 [33.25, 47.75] years versus (vs.) 27.00 [23.00, 34.00] years; *p* < 0.001). There was no statistical difference in the disease course between the two groups (3.00 [1.00, 12.00] months vs. 10.00 [2.00, 24.00] months; *p* = 0.059). Both groups were prone to abdominal pain (80.0% vs. 79.9%; *p* > 0.99), but diarrhea was more common in CD patients (35.0% vs. 76.5%; *p* < 0.001). Gastrointestinal bleeding, fever, and weight loss were more common in CD but not statistically significant. Oral and genital ulcers were more common in intestinal BD patients (80.0% vs. 5.6%, 40.0% vs. 0%; both *p* < 0.001). CD patients were more often presented with intestinal manifestation as an initial symptom (30.0% vs. 93.3%; *p* < 0.001). Perianal lesions were found in one hundred and nine CD patients, but none were found in intestinal BD patients (*p* < 0.001).

### 3.2. Laboratory Results

Intestinal BD patients were often presented with lower C-reactive protein (CRP) (11.50 [2.48, 34.93] mg/L vs. 25.00 [10.50, 48.00] mg/L; *p* = 0.014), erythrocyte sedimentation rate (ESR) (16.00 [10.00, 28.75] mm/h vs. 23.00 [13.00, 40.00] mm/h; *p* = 0.149), and hemoglobin (Hb) (113.80 ± 22.00 g/L vs. 115.58 ± 22.66 g/L; *p* = 0.739). However, intestinal BD patients had higher albumin (39.33 ± 6.41 g/L vs. 37.20 ± 6.54 g/L; *p* = 0.168).

### 3.3. Endoscopic Characteristics

**Figure 4:**
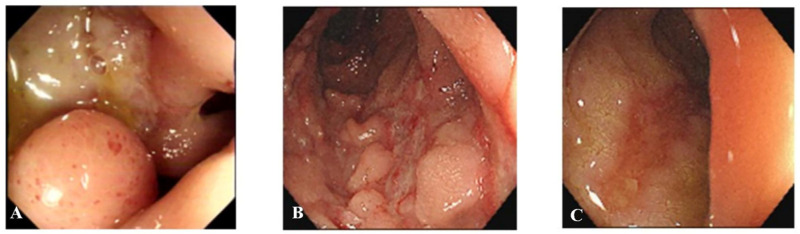
Endoscopic characteristics of intestinal BD and CD patients. (**A**) A large ulcer with a discrete margin was observed in the terminal ileum in an intestinal BD patient. (**B**,**C**) Typical longitudinal ulcer was seen in ascending and descending colon in CD patient. BD, Behçet’s disease; CD, Crohn’s disease.

Types of colonoscopies performed on patients were as follows: intestinal BD—small intestine endoscopy *(n* = 3), colonoscopy (*n* = 18); and CD—small intestine endoscopy (*n* = 40), colonoscopy (*n* = 145). Intestinal BD patients usually presented focal ulcer distribution (75.0% vs. 28.5%, *p* = 0.001), but still presented segmental involvement (25.0%). The ulcers of CD patients were longitudinal in shape (5.0% vs. 43.6%, *p* < 0.001) (Figure 4). Ileocecal valve deformity was presented more often (45.0% vs. 31.3%, *p* = 0.215) in intestinal BD patients. Although noncaseating granuloma was detected in only 56.2% CD patients during biopsy, this proportion remained considerably higher compared to those intestinal BD patients (10.0%).

### 3.4. Image Features

All image features except the pattern of bowel wall thickening assessed by the two senior radiologists reached excellent agreement. Therefore, in the subsequent model construction, among the image features with *p* < 0.05, we selected the top three with the highest consistency to be included in the comprehensive model. The specific results of the image features were shown in Table 2. Jejunum, proximal ileum, terminal ileum, and colorectal involvement were more common in CD patients (5.0% vs. 9.5%, *p* = 0.799; 5.0% vs. 45.8%, *p* < 0.001; 60.0% vs. 75.4%, *p* = 0.137; 45.0% vs. 65.9%, *p* = 0.065). Cecal involvement was more common in intestinal BD patients (80.0% vs. 66.5%, *p* = 0.220) and were more presented with polypoid thickening pattern (50.0% vs. 10.6%, *p* < 0.001). The enhancement pattern of the two groups was also different (homogeneous: 45.0% vs. 31.3%, layered: 55.0% vs. 68.7%, *p* = 0.314). The asymmetrical thickening of the bowel wall and the comb sign were more common in CD patients (15.0% vs. 41.3%, *p* = 0.022; 30.0% vs. 74.9%, *p* < 0.001). Intestinal BD patients had a higher rate of intestinal penetration (15.0% vs. 6.1%, *p* = 0.314), but lower rate of intestinal stenosis (15.0% vs. 44.7%, *p* = 0.011). 

### 3.5. Body Composition Analysis

The specific results of body composition analysis are shown in Table 3. The VSRL1-5 of CD patients was higher than in intestinal BD patients, but statistical differences were only found at the L4 and L5 levels (0.68 [0.37, 0.96] vs. 0.81 [0.54, 1.40], *p* = 0.046; 0.59 [0.36, 0.95] vs. 0.85 [0.54, 1.40], *p* = 0.013). VSR_volume_ was 0.77 (0.36, 1.11) in intestinal BD patients, and 0.97 (0.63, 1.35) in CD patients (*p* = 0.072). Based on VAT at five levels, we calculated the CV and found a higher value in CD patients (0.23 [0.19, 0.44] vs. 0.35 [0.25, 0.51], *p* = 0.039). As for the of attenuation of VAT, the quartile was found higher in CD patients at five levels (all *p* < 0.05).

### 3.6. Identification Models

**Figure 5:**
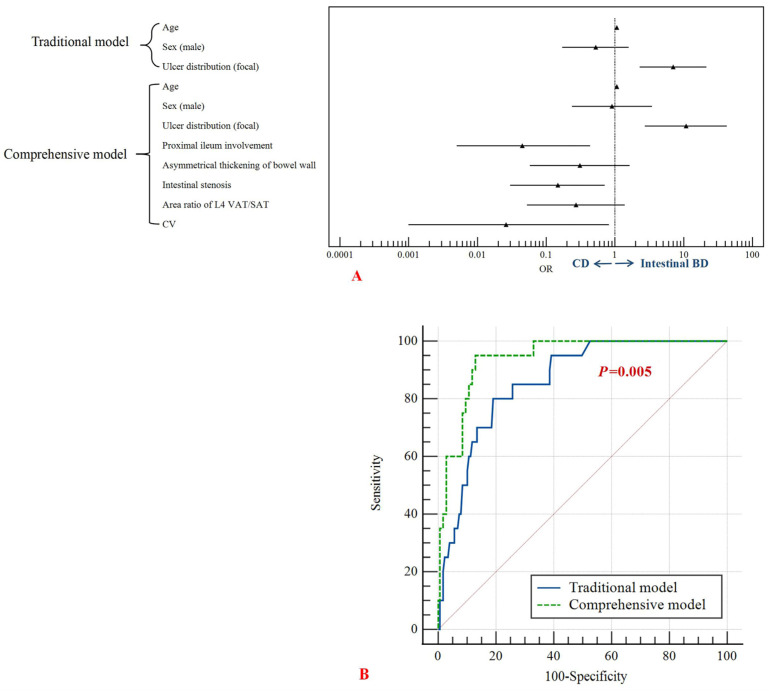
(**A**) The forest plot of the multivariate analysis results of the traditional model (age, sex and ulcer distribution) and the comprehensive model (age, sex, ulcer distribution, proximal ileum involvement, asymmetrical thickening of bowel wall, intestinal stenosis, area ratio of L4 VAT/SAT, and CV). OR > 1 favored the diagnosis of intestinal BD, while OR < 1 favored the diagnosis of CD. (**B**) The ROC curves of traditional model and comprehensive model. The sensitivity and specificity of the traditional model were 80.0% and 81.0%, respectively, with an AUC of 0.862, 95% CI: 0.792–0.933. The sensitivity and specificity of the comprehensive model were 95.0% and 87.2%, respectively, with an AUC of 0.941, 95% CI: 0.901–0.981. VAT, visceral adipose tissue; SAT, subcutaneous adipose tissue; CV, coefficient of variation; OR, odds ratio; BD, Behçet’s disease; CD, Crohn’s disease; ROC, receiver operating characteristic; AUC, area under the curve; CI, confidence interval.

The variables used in univariable analysis were age, sex, ulcer distribution, proximal ileum involvement, asymmetrical thickening of bowel wall, intestinal stenosis, VSR_L4_ and CV, and all *p* values were found to be <0.1 (Table S1). The above variables were included in the multivariable analysis to build two identification models (traditional model: age, sex, ulcer distribution; comprehensive model: age, sex, ulcer distribution, proximal ileum involvement, asymmetrical thickening of bowel wall, intestinal stenosis, VSR_L4_ and CV) (Table 4 and Figure 5). The area under curve (AUC) of the traditional (sensitivity: 80.0%, specificity: 81.0%) and comprehensive (sensitivity: 95.0%, specificity: 87.2%) models were 0.862 (95% confidence interval (CI): 0.792–0.933) and 0.941 (95%CI: 0.901–0.981), respectively (*p* = 0.005) (Figure 5). After conducting leave-one-out cross-validation, the traditional model achieved an AUC of 0.840, while the comprehensive model achieved an AUC of 0.912.

## 4. Discussion

The addition of CTE characteristics, including image features and body composition analysis in the present study, has significantly improved diagnostic accuracy compared to traditional approach, which solely relied on clinical features and endoscopic findings.

A study showed that more than half of intestinal BD patients were misdiagnosed as CD in the first hospitalization [22]. Therefore, it is crucial to find features that help distinguish intestinal BD from CD. At present, there was a scarcity of studies focusing on the discriminative features, and the sensitivity and specificity of identification based on different indicators display considerable variation [10,17,22,23,24]. Lee et al. developed a model based on endoscopic findings, including the shape, distribution, and number of ulcers, aphthous lesion and cobblestone appearance present [25]. The sensitivity and specificity were 94.3% and 90.0%, respectively. Longitudinal ulcers supported the diagnosis of CD and focal distribution supported the diagnosis of intestinal BD, which was consistent with our results. However, the accurate identification of ulcer shapes largely depends on the proficiency of the performing physician. On the other hand, the ulcer distribution only required recording the segment of the intestine where the ulcer was located, offering a more objective assessment. Regarding the malformation of the ileocecal valve, studies [10,17,23] presented different opinions. Jing-Fen Y.E. et al. (31/111 vs. 19/81, *p* = 0.486) and Ji Li et al. (16/35 vs. 16/106, *p* = 0.003) found a higher rate of malformation in the ileocecal valve in intestinal BD patients compared to in CD patients, which was consistent with our result. While Tianyu Zhang et al. found a different result (8/42 vs. 19/97, *p* = 0.941). Hence, the scientific research significance of ileocecal valve malformation remains uncertain.

A study [23] built a differential model based on endoscopy, and the AUC was 0.874. The clinical characteristics of intestinal BD and CD patients were also recorded; however, there was a lack of integration between clinical and endoscopic characteristics. The diagnosis of a disease usually needs a combination of multiple types of evidence. Hong Yang et al. [15] found that the model combined clinical manifestations and endoscopy demonstrated a higher diagnostic efficacy compared to solely relying on endoscopic characteristics. Almost all intestinal BD patients had recurrent oral ulcers. The incidence of genital ulcers with scarring was relatively low compared with oral ulcers. In present study, oral ulcers and genital ulcers were more common in intestinal BD patients, and most appeared before intestinal symptoms. Due to the inherent limitations of retrospective studies, biases can inadvertently influence medical records. For instance, when BD is strongly suspected clinically, there is a higher likelihood of selectively recording symptoms like oral and genital ulcers. In comparison, demographic data, such as age and sex, offer a general reference for disease characteristics and a higher degree of objectivity.

Therefore, based on clinical and endoscopic results and aforementioned discussion, we chose to incorporate age, sex, and ulcer distribution to construct a traditional identification model. However, in certain scenarios, intestinal stenosis can impede the insertion of an endoscope, leading to a partial observation of lesions across intestinal segments. Consequently, CTE has gained prominence as a crucial imaging modality for evaluating diverse intestinal conditions. In the present study, we observed a higher proportion of layered enhancement in the bowel wall and a higher CRP in CD patients. This suggested that CD patients tended to exhibit signs of active inflammation during their initial hospitalization. On the other hand, almost half of intestinal BD patients progressed into a chronic disease course. BD is a vasculitis disorder that can affect any blood vessels in the gastrointestinal tract, leading to intestinal ischemia and intestinal infarction. The main involvement was typically concentrated in the ileocecal region, which was similar to CD. But, CD patients tended to have a broader range of affected areas. In our study, the presence of proximal ileal involvement often supported the diagnosis of CD. Furthermore, the wall thickening observed in CD patients commonly displays an asymmetric pattern, primarily due to the preferential involvement along the mesenteric border of the bowel wall [26]. However, the specific mechanisms remain unclear. Furthermore, in our analysis of the disease behavior, due to the repeated inflammation and repair of the intestinal wall, CD patients often develop inflammatory and fibrous stenosis [27].

CTE can not only serve as an indicator for intestinal imaging but also facilitate body composition analysis. Research focusing on VAT in CD patients has attracted increasing interest. Nevertheless, there is a noticeable absence of studies investigating VAT in intestinal BD patients. Numerous studies have been conducted to identify CD and other similar diseases using VAT as a diagnostic tool [28,29]. Saurabh et al. found that VSRL4 in CD patients was higher than that of intestinal tuberculosis patients (*p* < 0.001), which was used to establish a differential diagnosis model for CD and intestinal tuberculosis. We analyzed VSR at five levels. Although the VSR of CD was higher in all five levels, only L4 and L5 presented statistical differences (*p* = 0.046, *p* = 0.013). In addition, unlike previous studies that primarily compared the VAT area of each level, we employed the CV for VAT assessment to reflect the spatial distribution. Notably, our findings revealed a significantly higher CV of VAT in CD patients, signifying a more heterogeneous distribution of VAT within this patient group. Since CD is a transmural inflammatory disease, the attenuation of VAT would then experience significant elevation. Previous studies calculated the average attenuation of VAT to predict outcomes in CD [30]; however, using the average will obscure distinctive characteristics. Our research focused on the attenuation distribution of VAT at five levels. Remarkably, our findings revealed that each quartile of VAT attenuation in CD patients surpassed the corresponding levels in intestinal BD patients. This distinction further bolsters the notion of more extensive and pronounced intestinal inflammation in CD patients compared to those with intestinal BD. Hence, by incorporating CTE characteristics such as proximal ileum involvement, the asymmetrical thickening of the bowel wall, intestinal stenosis, VSRL4, and CV, we augmented the traditional model and developed a new comprehensive framework. This yielded a notable improvement in the AUC, increasing from 0.862 to 0.941.

There are some limitations to our study. Firstly, the relatively low incidence of intestinal Behçet’s disease contributed to a small sample size of patients. To overcome this limitation in future follow-up research, we are considering the inclusion of patients with vasculitis to augment our sample. Additionally, while our study design was retrospective, we made careful choices of objective and highly consistent indicators to establish our models. Thirdly, we only performed leave-one-out cross-validation on the included patients. We did not have new cases for internal or external validation.

## 5. Conclusions

Intestinal BD and CD can be distinguished from each other. Compared with the traditional clinical plus endoscopy model, the inclusion of CTE can substantially enhance the effectiveness of differentiation.

## Figures and Tables

**Table 1:** The clinical information, laboratory results, and endoscopic characteristics of intestinal BD and CD patients.

Item	Intestinal BD (*n* = 20)	CD (*n* = 179)	*p* Value
Sex (male/female)	11/9	143/36	0.025
Age (year)	38.00 (33.25, 47.75)	27.00 (23.00, 34.00)	<0.001
Disease course (month)	3.00 (1.00, 12.00)	10.00 (2.00, 24.00)	0.059
Abdominal pain	16 (80.0%)	143 (79.9%)	>0.99
Diarrhea	7 (35.0%)	137 (76.5%)	<0.001
Fever	3 (15.0%)	36 (20.2%)	0.794
Weight loss	8 (40.0%)	83 (46.4%)	0.588
Gastrointestinal bleeding	11 (55.0%)	108 (60.3%)	0.644
Oral ulcer	16 (80.0%)	10 (5.6%)	<0.001
Genital ulcer	8 (40.0%)	0	<0.001
Intestinal symptoms as primary manifestation	6 (30.0%)	166 (93.3%)	<0.001
Perianal lesion	0	109 (60.9%)	<0.001
CRP (mg/L)	11.50 (2.48, 34.93)	25.00 (10.50, 48.00)	0.014
ESR (mm/h)	16.00 (10.00, 28.75)	23.00 (13.00, 40.00)	0.149
Albumin (g/L)	39.33 ± 6.41	37.20 ± 6.54	0.168
Range of albumin	22.5–50.6	21.2–67.9	
Hemoglobin (g/L)	113.80 ± 22.00	115.58 ± 22.66	0.739
Endoscope type			
Small intestine endoscopy	3	40	
Colonoscopy	18	145	
Longitudinal ulcer	1 (5.0%)	78 (43.6%)	<0.001
Ulcer distribution			0.001
Focal	15 (75.0%)	51 (28.5%)	
Segmental	5 (25.0%)	128 (71.5%)	
Ileocecal valve deformity	9 (45.0%)	56 (31.3%)	0.215
Noncaseating granuloma	2 (10.0%)	100 (56.2%)	<0.001

BD, Behçet’s disease; CD, Crohn’s disease; CRP, C-reactive protein; ESR, erythrocyte sedimentation rate.

**Table 2:** Image features of intestinal BD and CD patients.

Item	Intestinal BD (*n* = 20)	CD (*n* = 179)	*p* Value	Kappa Value
Pattern of bowel wall thickening			<0.001	0.755
Polypoid	10 (50.0%)	19(10.6%)		
Homogeneous	10 (50.0%)	160 (89.4%)		
Enhancement pattern			0.314	0.824
Layered	11 (55.0%)	123 (68.7%)		
Homogeneous	9 (45.0%)	56 (31.3%)		
Intestinal segment involvement				
Jejunum	1 (5.0%)	17 (9.5%)	0.799	0.939
Proximal ileum	1 (5.0%)	82 (45.8%)	<0.001	0.878
Terminal ileum	12 (60.0%)	135 (75.4%)	0.137	0.884
Cecum	16 (80.0%)	119 (66.5%)	0.220	0.884
Colorectum	9 (45.0%)	118 (65.9%)	0.065	0.934
Ascending colon	9 (45.0%)	81 (45.3%)	0.983	0.980
Transverse colon	0	77 (43.0%)	<0.001	0.958
Descending colon	0	67 (37.4%)	0.001	0.966
Sigmoid colon	0	72 (40.2%)	<0.001	0.968
Rectum	0	55 (30.7%)	0.004	0.889
Asymmetrical thickening of bowel wall	3(15.0%)	74 (41.3%)	0.022	0.836
Comb sign	6 (30.0%)	134 (74.9%)	<0.001	0.803
Intestinal stenosis	3 (15.0%)	80 (44.7%)	0.011	0.813
Penetration (sinus, fistula, abscess)	3 (15.0%)	11 (6.1%)	0.314	0.802

BD, Behçet’s disease; CD, Crohn’s disease.

**Table 3:** The specific results of body composition analysis.

Item	Intestinal BD (*n* = 20)	CD (*n* = 179)	*p* Value
VSR_L1_	0.86 (0.43, 1.78)	1.26 (0.59, 3.51)	0.086
VSR_L2_	0.91 (0.37, 1.47)	1.11 (0.64, 2.00)	0.063
VSR_L3_	0.75 (0.35, 1.17)	0.91 (0.37, 1.47)	0.088
VSR_L4_	0.68 (0.37, 0.96)	0.81 (0.54, 1.40)	0.046
VSR_L5_	0.59 (0.36, 0.95)	0.85 (0.54, 1.40)	0.013
VSR_volume_	0.77 (0.36, 1.11)	0.97 (0.63, 1.35)	0.072
CV	0.23 (0.19, 0.44)	0.35 (0.25, 0.51)	0.039
L1 VAT attenuation (HU)			
25% quartile	−101.65 ± 14.84	−90.18 ± 16.41	0.003
Median	−80.50 (−100.75, −70.00)	−68 (−82.00, −54.00)	0.003
75% quartile	−57.00 (−78.25, −41.50)	−41.00 (−54.00, −28.00)	0.001
L2 VAT attenuation (HU)			
25% quartile	−100.70 ± 12.77	−90.69 ± 17.49	0.014
Median	−82.15 ± 15.18	−69.91 ± 18.98	0.006
75% quartile	−56.50 (−75.75, −43.19)	−41.00 (−54.00, −29.00)	0.001
L3 VAT attenuation (HU)			
25% quartile	−103.55 ± 12.89	−93.38 ± 16.86	0.010
Median	−84.13 ± 15.71	−71.84 ± 19.28	0.003
75% quartile	−60.50 (−77.25, −44.75)	−43.00 (−57.00, −30.00)	0.002
L4 VAT attenuation (HU)			
25% quartile	−106.90 ± 9.71	−93.48 ± 17.70	0.001
Median	−88.75 ± 12.69	−71.80 ± 20.10	<0.001
75% quartile	−68.00 (−77.50, −53.00)	−42.00 (−58.00, −29.00)	<0.001
L5 VAT attenuation (HU)			
25% quartile	−101.70 ± 13.29	−90.13 ± 18.11	0.006
Median	−83.15 ± 16.15	−69.19 ± 20.39	0.003
75% quartile	−59.50 (−79.75, −45.50)	−42.00 (−58.00, −28.00)	0.001

CV means the coefficient of variation of the area of VAT of L1–L5. BD, Behçet’s disease; CD, Crohn’s disease; VSR, area ratio of visceral adipose tissue: subcutaneous adipose tissue; CV, coefficient of variation; VAT: visceral adipose tissue; HU, Hounsfield unit.

**Table 4:** Multifactor analysis of traditional model and comprehensive model.

Item	Traditional Model			Comprehensive Model		
	*p* Value	OR	95% CI	*p* Value	OR	95% CI
Age	0.002	1.069	1.025–1.116	0.016	1.060	1.011–1.112
Sex (male)	0.253	0.523	0.172–1.589	0.887	0.908	0.239–3.456
Ulcer distribution (focal)	0.001	7.020	2.290–21.524	0.001	10.772	2.738–42.383
Proximal ileum involvement				0.007	0.045	0.005–0.435
Asymmetrical thickening of bowel wall				0.167	0.308	0.058–1.637
Intestinal stenosis				0.017	0.147	0.030–0.710
Area ratio of L4 VAT/SAT				0.118	0.271	0.053–1.391
CV				0.038	0.026	0.001-0.822

CV means the coefficient of variation of the area of VAT of L1–L5. OR > 1 favored the diagnosis of intestinal BD, while OR < 1 favored the diagnosis of CD. OR, odds ratio; CI, confidence interval; VAT, visceral adipose tissue; SAT, subcutaneous adipose tissue; CV, coefficient of variation.

## Data Availability

All data are available in this article.

## Supplementary Materials

The following supporting information can be downloaded at: https://www.mdpi.com/article/10.3390/bioengineering10101211/s1, Table S1. Univariable analysis for the variables of interest.
