# Supplementary material for: Enhancing the Differentiation between Intestinal Behçet’s Disease and Crohn’s Disease through Quantitative Computed Tomography Analysis
Source: Bioengineering (Basel). 2023 Oct 17;10(10):1211. doi: 10.3390/bioengineering10101211 (PMC10604024; doi:10.3390/bioengineering10101211)
Supplement: Supplementary file 1 [file bioengineering-10-01211-s001.zip › bioengineering-2624929-supplementary.pdf]

Table S1. Univariable analysis for the variables of interest.

Item	*p* Value	OR	95% CI
Age	<0.001	1.076	1.037-1.117
Sex (male)	0.015	0.308	0.119-0.799
Ulcer distribution (focal)	<0.001	7.529	2.601-21.795
Proximal ileum involvement	0.007	0.062	0.008-0.475
Asymmetrical thickening of bowel wall	0.032	0.250	0.071-0.885
Intestinal stenosis	0.018	0.218	0.062-0.772
Area ratio of L4 VAT/ SAT	0.056	0.287	0.080-1.030
CV	0.058	0.060	0.003-1.103

CV means the coefficient of variation of the area of VAT of L1-L5.

OR, odds ratio; CI, confidence interval; VAT, visceral adipose tissue; SAT, subcutaneous adipose tissue; CV, coefficient of variation.
